# Microbiome differences in disease-resistant vs. susceptible *Acropora* corals subjected to disease challenge assays

**DOI:** 10.1038/s41598-019-54855-y

**Published:** 2019-12-04

**Authors:** Stephanie M. Rosales, Margaret W. Miller, Dana E. Williams, Nikki Traylor-Knowles, Benjamin Young, Xaymara M. Serrano

**Affiliations:** 1 Atlantic Oceanographic and Meteorological Laboratory, National Oceanographic and Atmospheric Administration, Miami, Florida USA; 2 Cooperative Institute for Marine and Atmospheric Studies, University of Miami, Miami, Florida USA; 3 SECORE International, Miami, FL 33145 USA; 4 Southeast Fisheries Science Center, NOAA-National Marine Fisheries Service, Miami, FL USA; 5 University of Miami, Rosenstiel School of Marine and Atmospheric Sciences, Miami, USA

**Keywords:** Microbiome, Bacterial genes

## Abstract

In recent decades coral gardening has become increasingly popular to restore degraded reef ecosystems. However, the growth and survivorship of nursery-reared outplanted corals are highly variable. Scientists are trying to identify genotypes that show signs of disease resistance and leverage these genotypes in restoring more resilient populations. In a previous study, a field disease grafting assay was conducted on nursery-reared *Acropora cervicornis* and *Acropora palmata* to quantify relative disease susceptibility. In this study, we further evaluate this field assay by investigating putative disease-causing agents and the microbiome of corals with disease-resistant phenotypes. We conducted 16S rRNA gene high-throughput sequencing on *A. cervicornis* and *A. palmata* that were grafted (inoculated) with a diseased *A. cervicornis* fragment. We found that independent of health state, *A. cervicornis* and *A. palmata* had distinct alpha and beta diversity patterns from one another and distinct dominant bacteria. In addition, despite different microbiome patterns between both inoculated coral species, the genus *Sphingomonadaceae* was significantly found in both diseased coral species. Additionally, a core bacteria member from the order Myxococcales was found at relatively higher abundances in corals with lower rates of disease development following grafting. In all, we identified *Sphingomonadaceae* as a putative coral pathogen and a bacterium from the order Myxococcales associated with corals that showed disease resistant phenotypes.

## Introduction

To mitigate the loss of coral reefs and their genetic diversity, coral restoration practices have increased in the past decade^1,2^. As a form of active restoration, corals are propagated by “coral gardening” in ocean nurseries and then outplanted onto degraded reefs^3^. Across the Caribbean, restoration programs have outplanted thousands of corals in degraded coral reef systems^1^. Coral gardening has become increasingly popular and successful, especially for acroporid corals due to their fast growth rates^4^. However, there are varying levels of success rates in acroporid outplant because they are exposed to different environments^5^, heat stress^6^, and diseases^7^. The ability for these outplants to withstand these different stressors is often attributed to a coral genotype effect^5–7^.

The corals *Acropora cervicornis* and *Acropora palmata* were historically the dominant species in the Caribbean but are now both critically endangered^8,9^. Their populations have plummeted mostly due to disease outbreaks^10^ and heat stress caused by global climate change^11^. The severity of both climate change and disease outbreaks is predicted to increase in the coming years^12^. Therefore, outplanted *Acropora spp*. face similar (or more severe) environmental challenges to those that contributed to their initial population decline^6,7^.

To improve restoration efforts, researchers are investigating traits of coral resilience to significant stressors such as disease. In natural populations of *A. cervicornis*, *in situ* transmission assays of white-band disease (WBD) showed that 6% of genotypes were resistant to disease^13^. In lab, WBD homogenate challenges on *A. cervicornis* nursery corals also resulted in distinct responses among genotypes^14^. In addition, inoculation with a *Vibrio* pathogen also caused differences in disease susceptibility between *Acropora millepora* genotypes and a shift in the microbial community to a more pathogenic state^15^.

A shift in coral microbiomes after a disease exposure is well documented in the literature and has recently been reviewed^16^. However, it has been proposed that microbes also play a role in coral resilience^16–19^. Coral microbes are essential contributors to the homeostasis of the coral host and pathogen control^16^. For example, when the coral *Montastraea cavernosa* was simultaneously inoculated with the pathogen *Vibrio coralliilyticus* and a core microbiome member, *Halobacteriovorax*, there was a reduction in *Vibri*o and opportunistic pathogens^19^. In a similar study, putatively beneficial microorganisms (pBMCs) were shown to reduce the effects of *Vibrio* and bleaching^20^.

Recently, researchers characterized disease susceptibility among genotypes from both *A. cervicornis* and *A. palmata* from three coral nurseries across two years (in July–Sept 2016 and July–Aug 2017)^21^. In the study, they found that coral genotypes showed significant variations when visually healthy corals were grafted to diseased ramets (the “inoculants”). Transmission to apparently healthy corals was identified phenotypically by the appearance of tissue-loss lesions. Diseases in the field are often difficult to characterize because of the shared physical signs that diseases manifest in corals^22^. However, the display of disease signs following exposure to a diseased fragment means that the apparently healthy coral was likely infected by a pathogen that was transmitted from the diseased coral. Several such agents have been associated with tissue-loss disease in acroporids^7,23^.

Histological investigations from 11 corals suggested that in 2016 the inoculant corals may have been affected by WBD^21^. In 2017, histology results on the inoculants (N = 3) showed features distinct from the majority of 2016 samples that were consistent with rapid tissue loss (RTL)^21^. In 2017, inoculated corals were more susceptible to development of disease signs based on a higher risk of transmission^21^. This observation may have been because of a more virulent disease agent or because the specific genotypes tested in 2017 were less resistant to disease.

When characterizing disease susceptibility, it is useful to know which specific pathogens corals may be resistant against. Although a coral may be resistant to one pathogen, it does not necessarily mean that it will be resistant to a different pathogen. Here, we extended the study of Miller *et al*. (2019)^21^ by molecular characterization of the microbiome for a subset of 2017 samples. We aimed to: (1) characterize correlations between disease inoculated corals and their microbiome, (2) characterize putative disease agents transmitted to healthy corals, and (3) characterize the microbiome of corals with different disease susceptibility phenotypes.

## Methods

### Sample selection

In this study, we used corals that were collected in 2017 to characterize disease susceptibility among nursery stocks (from the Coral Restoration Foundation [CRF], Florida Fish and Wildlife Conservation Commission [FWC], and University of Miami [UM] nurseries; Table 1), as previously described^21,24^. Briefly, replicate ramets (or coral branches) from each tested genotype of *A. cervicornis* and *A. palmata* were grafted to *A. cervicornis* ramets that had visual disease signs (extended bright white exposed skeleton next to sharp or a jagged tissue margin). The ramets were monitored over a 7-day period and the appearance of tissue-loss lesions on a test ramet was taken as evidence of disease development following the grafting and hence, susceptibility. The percent of replicate ramets resulting in disease signs within the 7-day assay was used as a score of disease risk indicating relative disease susceptibility for that genotype.

**Table 1:** List of samples sequenced for this study.

Outcome				Control	Diseased	Visually Unaffected	Total
Coral Species	Genotype	Genotype name from nursery	Nursery				
*Acropora cervicornis*	C14	C1404	FWC		3		3
	C17	Kelsey	UM	2	4		6
	C18	KBCF-32	UM	2	1	2	5
	C20	Stag	UM	3	2	2	7
	C21	Elkhorn	UM	3	1		4
	C22	POM3	UM	2	2		4
	C24	Cooper-9	UM	2	1	3	6
	C28	C1398	FWC	3	2	3	8
	C29	Genet21	FWC	3	1	4	8
	C30	Genet23	FWC	3	1	5	9
*Acropora palmata*	P7	CN4	CRF	3	1	2	6
	P8	ML2	CRF	3	3		6
	P9	SI5	CRF	3	2	1	6
	P10	SI1	CRF	3	3		6
	P11	AAA3	CRF	2	3		5
	P12	AAA2	CRF	3	1	2	6
				40	31	24	95

FWC = Florida Fish and Wildlife Conservation Commission, UM = University of Miami, and CRF = Coral Reef Foundation.

The in-field grafting experiments took place over two years (from July–Sept 2016 and July–Aug 2017). For this study, we focused on samples collected from the 2017 experiments, in which the disease appeared to be more virulent (i.e., rapid onset and more severe tissue loss)^21^. Tissue samples from each ramet were collected before grafting (Day 0), and after trials were completed (Day 7) by snipping a section of the branch base (which varied in distance from the lesion margin) into a labelled zip-top bag. On the boat, the snipped coral branch (which included skeleton, tissue, and mucus layers) were placed in a 2.0-mL cryovial with RNALater. Ninety-five samples were selected to match samples previously extracted for transcriptomic analysis (for an ongoing study). If the sample was depleted from the transcriptome extraction protocol then a tested sample from the same genotype was selected as a replacement. For *A. cervicornis*, there were 60 samples from 10 distinct genotypes (C14, C17, C18, C20, C21, C22, C24, C28, C29, and C30; Table 1), and in *A. palmata*, 35 samples were selected from 6 genotypes (P7, P8, P9, P10, P11, and P12; Table 1). The “Control” samples were samples taken from test ramets prior to grafting (0 d); “Diseased” samples showed tissue-loss lesions after a diseased ramet was grafted (7 d); and “Visually unaffected” corals showed no signs of tissue loss after a diseased ramet was grafted (7 d). Genotypes were also categorized as low, medium (mid), and highly susceptible to disease based on risk of transmission of disease signs (i.e., % of inoculated ramets that developed disease signs^21^). Genotypes that had <30% (C14, C24, C30), 40–60% (C18, C20, C29, C30) and >70% (C17, C21, C22) risk of transmission were considered to have low, mid, and high disease susceptibility, respectively. The high risk of transmission category constituted statistically significant (p < 0.05) higher disease-susceptible genotypes in the experiment^21^.

### DNA extractions and high-throughput amplicon sequencing

To extract DNA, coral tissue (~0.2 cm^2^) was first placed in a Power Bead Tube (Qiagen) and homogenized horizontally with a vortex adapter for 10 minutes. The samples were then processed using the DNeasy PowerSoil kit (Qiagen, Germantown, MD) following the manufacturer’s protocol. Extracted DNA was PCR amplified with 16S rRNA gene V4 primers^25^ using the Earth Microbiome Project (EMP) protocols^26^. Briefly, samples were processed using the Platinum Hot Start PCR Master Mix (2X) (ThermoFisher Scientific, Waltham, MA) in a 50-µl reaction with 2 µl of DNA template or 2 µl of PCR grade water for the negative control. DNA was amplified with the following parameters: 94 °C for 3 minutes (1X), 94 °C for 45 seconds (35X), 50 °C for 60 seconds (35X), 72 °C for 90 seconds (35X), and 72 °C for 10 minutes (1X). PCR products were cleaned using AMPure XP beads (Beckman Coulter, Brea, CA). Each cleaned, amplified, and barcoded DNA sample was normalized to 4 nM (except for the negative control due to its low DNA quantity). After, 5 µl of each sample and the PCR negative control were all pooled, and sent to the Hussman Institute for Human Genomics University of Miami Miller School of Medicine for sequencing on the MiSeq with PE-300v3 kits.

### Bioinformatic analysis

The sequences were demultiplexed by the core facility. The demultiplexed reads were then imported into Qiime2-2018.6^27^ and primers were trimmed using the program Cutadapt^28^. Forward reads were trimmed based on quality score decreases (phred score <30) at the first 10-bps and at the 200-bps position, and the reverse reads were trimmed at the first 10-bps and at the 110-bps position. The DADA2 program^29^ was used to cluster sequences by amplicon sequence variants (ASVs). The DADA2 pipeline was also used to quality filter (maxEE = 2), merge paired-ends, dereplicate, and remove chimeras. ASV’s were taxonomically classified with the Qiime2 feature-classifier classify-sklearn against the SILVA-132-99 database that was trained with a Naïve Bayes classifier on the 515–806 16S rRNA gene region. Reads with a taxonomical identification to mitochondria or chloroplasts were removed from further analysis. Upon initial analysis of the data, an ASV classified only to the kingdom level with a mean relative abundance of ~8% was identified. Further evaluation of this ASV with BLASTn®^30^ indicated that this was a mitochondria sequence. To further filter the data, reads classified only to the kingdom level were evaluated with BLASTn® against the non-redundant database. Twelve sequences with similarities (e-value = 0.001) to eukaryotes were removed.

### Statistical analysis

To analyze alpha-diversity, each coral species was evaluated together and separately. Sequences were rarefied to the minimum sequence depth found in each species when evaluated separately (*A. cervicornis* = 18,402 and *A. palmata* = 8,721) and to a minimum depth across samples (8,721) when evaluated together. To test the significance of Shannon diversity and evenness in host species, genotype, treatment (control and inoculated), and outcome (control, visually unaffected, and diseased) the alpha-group-significance function from Qiime2-2018.6 was used to test pairwise and all-group comparisons with a Kruskal-Wallis test.

For beta-diversity analysis, for each species, ASVs were removed if they did not appear in at least 4 samples. The abundance filtered count tables were imported into R v3.5.1 and converted into a phyloseq v1.26.1 object^31^. The count data were then transformed to centered log ratios (CLR) using microbiome v0.9.99^32^. The transformed matrix was then ordinated with a Euclidean distance and plotted on a Principal Components Analysis (PCA). The dispersion of the samples was tested by using the Vegan v2.5.4 package function vegdist (method = “Euclidean”, Permutations = 999) and betadisper^33^. An analysis of variance (ANOVA) was used to test the significance of dispersion across groups and a Tukey multiple comparison of means (function TukeyHSD) was used for pairwise comparisons. To statistically evaluate the significance of groupings an analysis of similarities (ANOSIM; distance = “Euclidean”, Permutations = 999) was used^34^. The interactions between groupings were tested with a Permutational multivariate analysis of variance (PERMANOVA; function Adonis, method = “Euclidean”, Permutations = 999)^34^. To assess microbial differential abundances, the abundance filtered count tables were analyzed with the analysis of composition of microbiomes (ANCOM) program^35^ on Qiime2. Each species was examined separately for differential abundance analysis against genotype, and experimental outcome. Statistical analysis was considered significant if they had a p-value < 0.05.

The core microbiome can be defined by a set of taxa shared across all or most of a particular habitat^36^. Bacteria taxa have been considered as part of the core if they are present in 30–100% of samples^37^. In this study, we analyzed the core microbiome for the categories “Outcome” and “Susceptibility.” In the outcome category, each species was parsed into subcategories by “Control,” “Diseased,” and “Visually Unaffected.” Only *A. cervicornis* samples showed significant differences in disease susceptibility by genotype^21^, thus only the core microbiome for *A. cervicornis “*Susceptibility” was examined. These samples were subdivided into susceptibility categories of, “Low,” “Mid,” and “High” based on transmission rates recorded in Miller *et al*.^21^. The core function from the microbiome package^32^ was used to determine which ASVs were present in at least 99% of samples (independent of abundance) from each subcategory. The mean, SD, minimum and maximum relative abundance for each core taxon within a subcategory was generated.

The metadata file and code for this study are available on https://github.com/srosales712/coral_disease_resistance_microbiome.git. The ASV sequences, feature count tables, and taxonomy files are available on figshare (10.6084/m9.figshare.8226209). The demultiplexed raw sequences can be found on NCBI’ Sequence Read Archive (SRA) under the BioProject ID: PRJNA546259. The field activity was conducted under permit number FKNMS-2014-047.

## Results

A total of 96 samples were processed (95 coral samples and one negative PCR control), but one *A. palmata* sample failed during sequencing. In total, 5,003 ASVs were identified. The minimum frequency of ASVs in a sample was 1,780 (from the negative control), the maximum frequency of ASVs in a sample was 583,256, and samples had a median ASV frequency of 160,275. After filtering, 192 and 225 ASVs remained for *A. cervicornis* (N = 60) and *A. palmata* (N = 35), respectively. For *A. cervicornis*, the minimum frequency for ASVs in a sample was 15,109 and the maximum was 546,739 with a median of 141,088. In *A. palmata*, the minimum ASV frequency in a sample was 8,560 and the maximum was 216,059 with a median of 68,560. The ASV frequency per sample is listed in the metadata file.

### The corals *A. cervicornis* and *A. palmata* had distinct microbial communities

**Figure 1:**
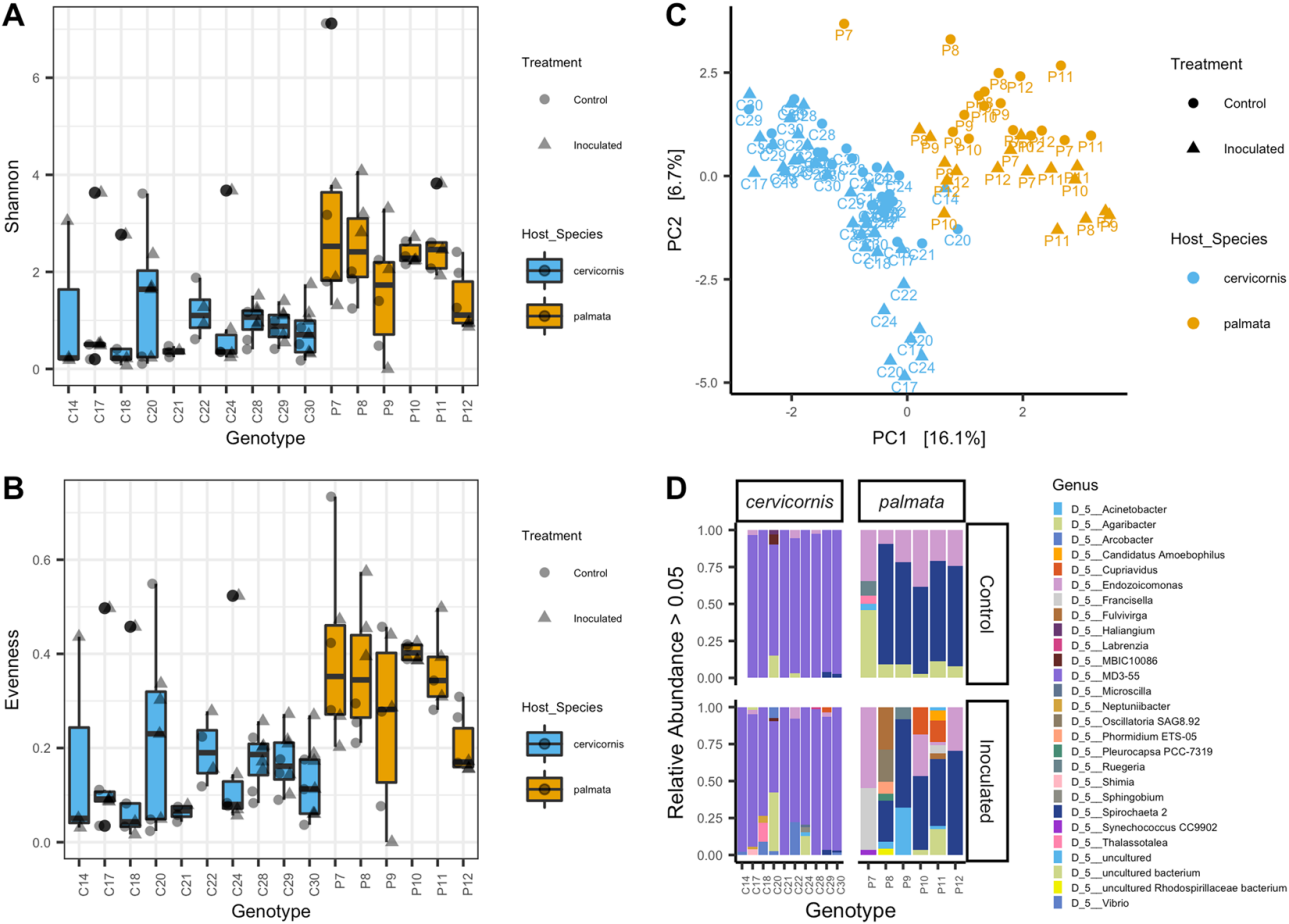
*A. cervicornis* and *A. palmata* show distinct microbial communities. There were significant differences between *A. cervicornis* and *A. palmata* in microbial (**A**) Shannon diversity (rarefied to 8,721) (**B**) evenness (rarefied to 8,721) and (**C**) beta-diversity (values were centered log ratio (CLR) transformed and plotted with a Euclidean distance on a principal component analysis (PCA) and only amplicon sequence variants (ASVs) present in >4 samples were used.). (**D**) The cumulative relative abundances of the most abundant microbial genera (>0.05%, not rarefied) per genotype. Each stacked color bar represents a different genus. The figure is grouped by coral species and treatment. For figures (**A**–**C**), circle = control samples, triangles = inoculated samples, blue = *Acropora cervicornis*, and tan = *Acropora palmata*.

When evaluated together, *A*. *cervicornis* and *A. palmata* were significantly different from one another in both alpha-diversity (Shannon; p-value = 5.02e^−9^ and evenness; p-value = 8.38e^−8^; Fig. 1A,B) and beta-diversity (ANOSIM p-value = 0.001, R^2^ = 0.47; Fig. 1C). The dominant bacteria members across all *A. cervicornis* samples belonged to the genus MD3-55 from the family *Midichloriaceae* (mean relative abundance (RA) = 51.82%, standard deviation (SD) = 34.07%, N = 60; Fig. 1D and Supplemental Fig. 1). This group was followed by an uncultured bacterial (i.e., has not been grown in culture) genus from the order Myxococcales (mean RA = 2.80%, SD = 13.23%; Fig. 1D and Supplemental Fig. 1). Combining all *A. palmata* samples, the dominant genus was *Spirochaeta* 2 from the family *Spirochaetaceae* (mean RA = 15.00%, SD = 28.00%; N = 35. Figure 1D and Supplemental Fig. 1), and the second dominant bacterium was *Endozoicomonas* (mean RA = 6.29%, SD = 16.79%; Fig. 1D and Supplemental Fig. 1). For the negative control there were 7 dominant bacterial genera with *Ralstonia* found at higher relative abundances (Supplemental Fig. 1A), but all bacteria were found at low relative abundances (Supplemental Fig. 1B).

### Microbial alpha-diversity was similar for genotypes from the same coral-host and increased when inoculated with a diseased coral

In both coral species microbial alpha diversity was not significantly different across coral genotypes, but there was a trend in alpha-diversity when samples were exposed to diseased fragments. In *A. cervicornis* alone, alpha-diversity for coral genotype and outcome (control, visually unaffected, and diseased) was not significantly different with both Shannon diversity and evenness metrics. In *A. cervicornis*, alpha-diversity significantly increased in inoculated samples compared to control samples (i.e., treatment) in Shannon diversity (p-value = 0.03) and evenness (p-value = 0.03). For *A. palmata*, genotype and treatment were not significant for Shannon diversity and evenness. Both alpha-diversity metrics were nearly significant for the experimental outcome (p-value = 0.055) of *A. palmata*, with visually unaffected and diseased corals showing higher diversity measurements compared to controls.

### The outcome from the grafting assay showed different microbiome beta-diversity patterns between Caribbean Acropora spp

**Figure 2:**
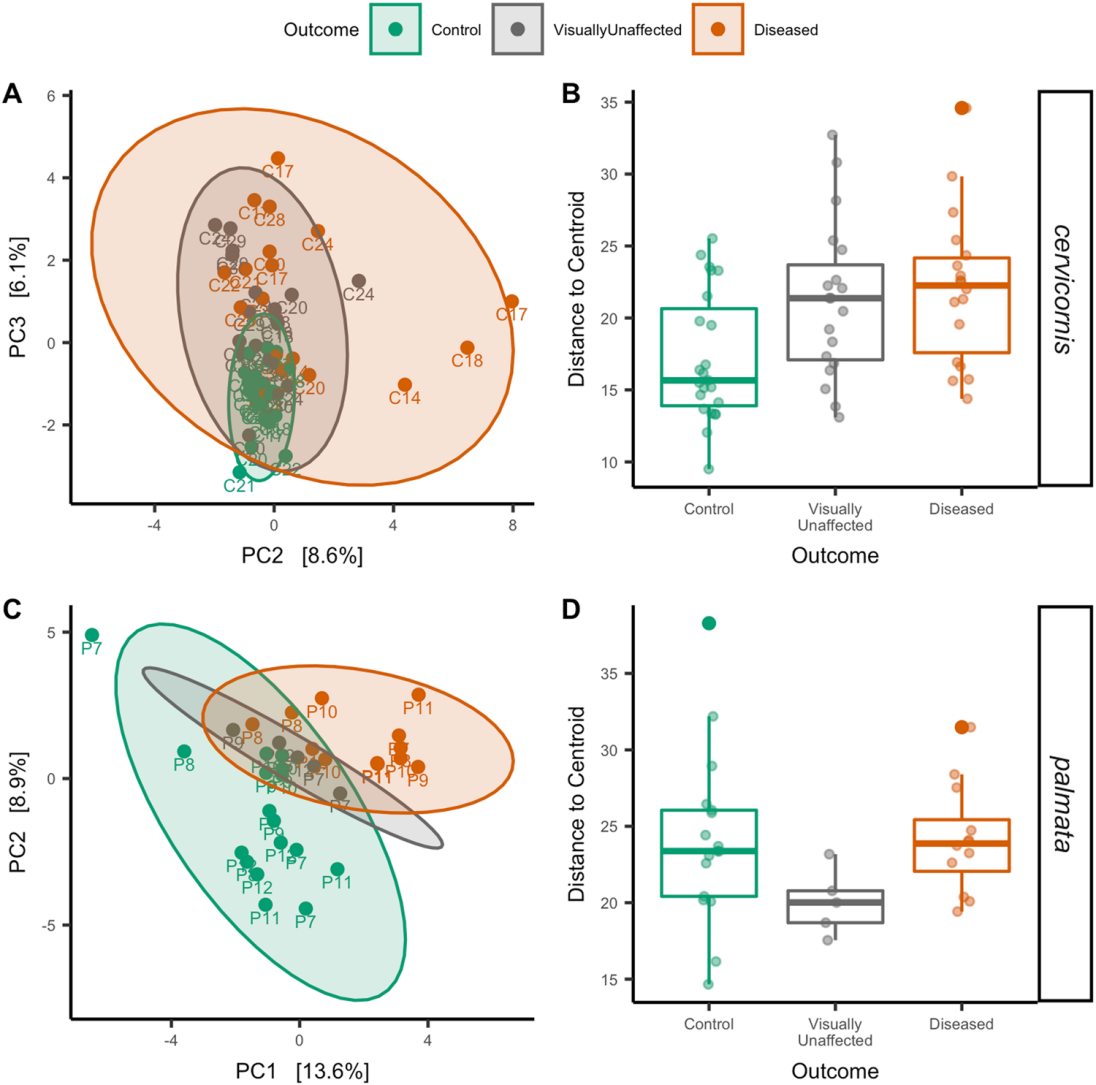
*A. cervicornis* and *A. palmata* show distinct beta-diversity patterns to disease exposure. (**A**) Principal component analysis (PCA) with a Euclidean distance of *A. cervicornis* colored by experiment outcome. (**B**) Dispersion of beta-diversity from the experiment outcome for *A. cervicornis*. (**C**) PCA with a Euclidean distance of *A. palmata* colored by experiment outcome (D) Dispersion of beta-diversity from the experiment outcome for *A. palmata*.

In *A. cervicornis*, beta-diversity was not a significant factor between treatments, but was significant for both genotype (ANOSIM; p-value = 0.001 R^2^ = 0.28; Supplemental Fig. 2A) and outcome (ANOSIM; p-value = 0.001 R^2^ = 0.17; Fig. 2A). The interaction between outcome and genotype was also significant (PERMANOVA; p-value = 0.030 R^2^ = 0.20). A beta-diversity dispersion analysis among genotypes resulted in no significant values. However, dispersion was significant for outcome (ANOVA; p-value = 0.007; Fig. 2C) and treatment (ANOVA; p-value = 0.0004). A pairwise comparison for outcome resulted in significance between not-exposed and no-transmission (TukeyHSD; p-value = 0.03; Fig. 2C) and not-exposed and transmission (TukeyHSD; p-value = 0.01; Fig. 2C). For *A. palmata*, beta-diversity groupings were significant for treatment (ANOSIM; p-value = 0.001 R^2^ = 0.19), genotype (ANOSIM; p-value = 0.001 R^2^ = 0.19; Supplemental Fig. 2B), and outcome (ANOSIM; p-value = 0.036 R^2^ = 0.14; Fig. 2B). Interactions between genotype-treatment and genotype-outcome were not significant for *A. palmata*. An analysis of dispersion also did not yield any significant results for treatment, outcome (Fig. 2D), or genotype.

### ASVs were significantly associated with coral genotypes and the outcome from the grafting assay

**Figure 3:**
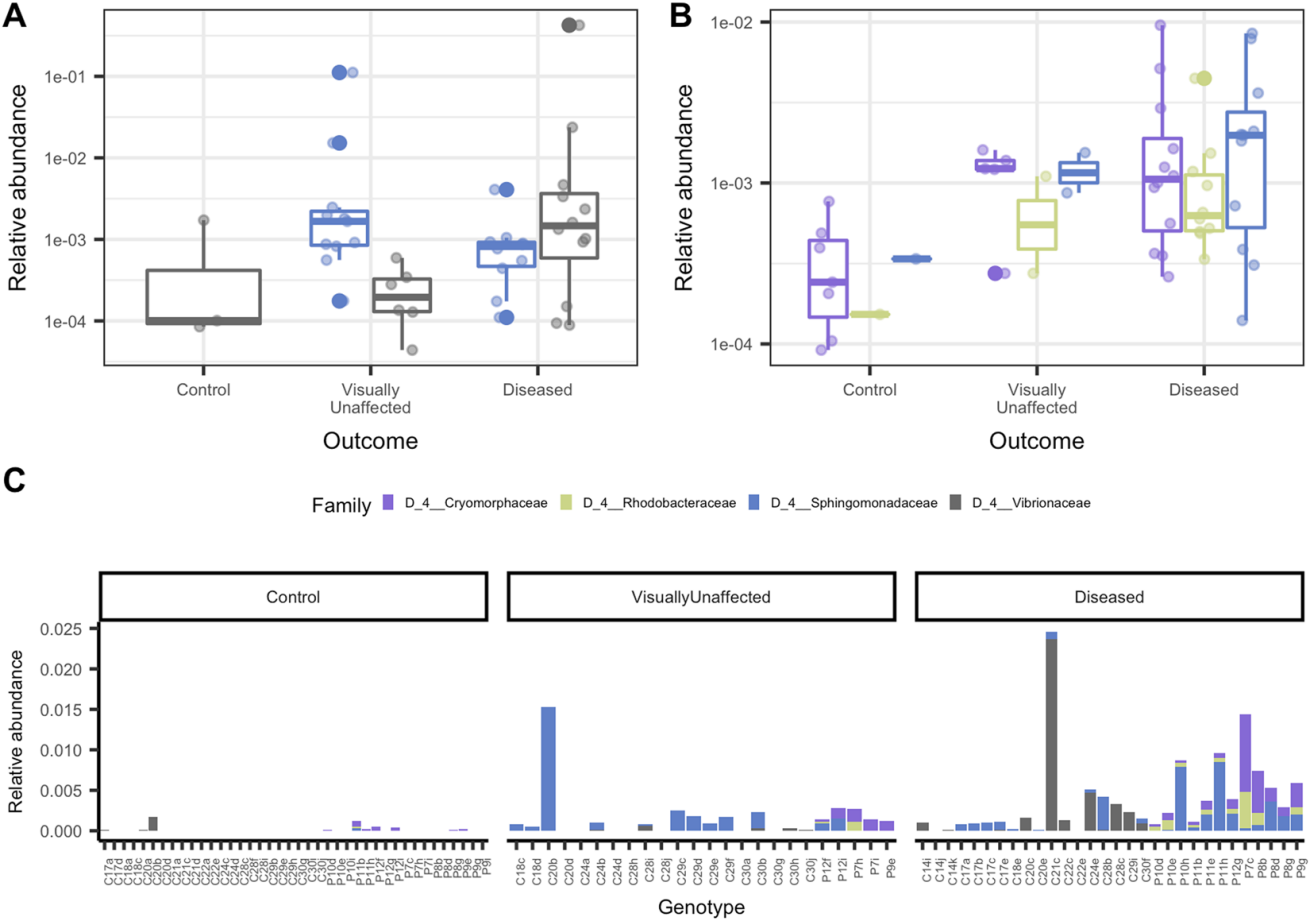
ASVs from four bacterial families were significantly associated with disease exposure. (**A**) Box plots show the relative abundance of a differential abundance analysis of the experiment outcome that resulted in two significantly abundant ASVs in *A. cervicornis*. (**B**) Differential abundance analysis of the experiment outcome resulted in three significantly abundant ASVs in *A. palmata*. (**C**) The average relative abundance of significantly differentiated ASVs per sample and grouped by outcome. For figures **A**–**C** bacterial families are represented by different colors.

A differential abundance analysis conducted to identify the differences across genotypes within a species resulted in five significant ASVs for *A. cervicornis* from the families *Spirochaetaceae, Endozoicomonadaceae, Francisellaceae*, and the order SAR324 clade (Supplemental Fig. 3A). Bacteria taxa that were significantly different in abundance among outcomes from the grafting assay in *A. cervicornis* was the species *Sphingobium yanoikuyae* and the genus *Vibrio* (Fig. 3A,C). Both these taxa were found at relatively higher abundances in visually unaffected and diseased corals compared to control corals. In *A. palmata* corals, three bacterial taxa were significantly different within genotypes and belonged to the families *Spirochaetaceae*, and *Endozoicomonadaceae* (Supplemental Fig. 3B). For outcome in *A. palmata*, the species *S. yanoikuyae* and the families *Rhodobacteraceae* (genus HIMB11) and *Cryomorphaceae* (uncultured genus) were significantly abundant and more highly associated with visually unaffected and diseased corals (Fig. 3B,C). In both coral species, *S. yanoikuyae* was identified as significantly different in abundance and had a mean RA of 3.03 × 10^−1%^ (SD = 1.86% min. = 0.01%, and max = 11.16%) and 0.12% (SD = 0.24%, min. = 0.01%, and max = 0.85%), for *A. cervicornis* and *A. palmata*, respectively. *S. yanoikuyae* was present in 67% (21/31) of diseased samples, in 54% (13/24) of visually unaffected samples and in one control sample (1/40) (Fig. 3C).

### The core microbiome may have a role in disease susceptibility

We defined the core microbiome as taxa present in 99% of samples from a group or category, independent of abundance. The core microbiomes identified along with the mean, SD, min, and max relative abundance are listed in Tables 2 and 3. In *A. cervicornis* the core microbiome for control samples (N = 23) resulted in 4 core taxa, visually unaffected samples (N = 19) also had 4 core taxa, and diseased (N = 18) samples had 5 core taxa (Table 2). When combined there was a total of 6 unique core taxa across the samples. The core microbiome of *A. palmata* resulted in 3 core taxa for controls (N = 17), 5 core taxa for visually unaffected samples (N = 5), and 6 core taxa in diseased samples (N = 12). Combined, there was a total of 10 unique core taxa or ASVs in *A. palmata*. Within the disease category, the family *Cyanobiaceae* was present in the disease core microbiome of both coral species. Thus, the relative abundance of this ASV was plotted to identify patterns of disease, but this ASV was also present in control samples at similar abundances (data not shown). For *A. cervicornis* genotypes within low (N = 18; C14, C24, and C30), mid (N = 28; C18, C20, C28, and C29), and high (N = 14; C17, C21, and C22) susceptibility subcategories there were 5, 4, and 8 core taxa bacteria identified (Table 3). A total of 8 unique ASVs resulted when the subcategories were combined.

**Table 2:** Relative abundance (RA) by taxon of core microbiomes per experimental outcome and coral-host. Percentages were generated by aggregating ASVs at the family level. ASVs that were not classified to the family level are listed to the lowest taxonomic classification.

Species	Outcome	Taxon	%Mean RA	%SD RA	%Min RA	%Max RA
*Acropora cervicornis*	Control (N = 23)	*Midichloriaceae*	93.2	18.6	8.7	99.8
		*P3OB-42*	4.6	17.7	0.01	85.5
		*Endozoicomonadaceae*	2.2	2.6	0.02	10.0
		Proteobacteria	0.03	0.03	0.009	0.2
	Visually unaffected (N = 19)	*Midichloriaceae*	83.6	33.1	6.3	99.9
		*P3OB-42*	14.2	32.9	0.008	92.0
		*Endozoicomonadaceae*	2.2	2.9	0.009	11.0
		Proteobacteria	0.03	0.02	0.01	0.09
	Diseased (N = 18)	*Midichloriaceae*	97.4	3.9	82.9	99.8
		*Endozoicomonadaceae*	1.3	3.6	0.04	15.4
		*P3OB-42*	0.8	1.2	0.01	3.9
		*Helicobacteraceae*	0.3	0.3	0.02	1.4
		*Cyanobiaceae*	0.1	0.1	0.02	0.5
*Acropora palmata*	Control (N = 17)	*Spirochaetaceae*	65.1	37.4	0.18	99.7
		*Endozoicomonadacea*e	26.0	33.4	0.020	99.2
		*Midichloriaceae*	8.8	23.2	0.2	71.8
	Visually unaffected (N = 5)	*Spirochaetaceae*	57.0	51.3	0.4	97.0
		Proteobacteria	21.4	42.5	0.4	97.2
		*P3OB-42*	17.6	39.0	0.09	87.4
		*Midichloriaceae*	2.8	2.5	0.2	6.6
		*Cryomorphaceae*	0.76	1.23	0.1	3.0
		*Vibrionaceae*	0.30	0.3	0.08	0.7
		*Phycisphaeraceae*	0.12	0.13	0.01	0.3
	Diseased (N = 12)	*Spirochaetaceae*	79.2	29.1	2.5	98.9
		*Endozoicomonadaceae*	13.1	18.1	0.07	54.6
		*Midichloriaceae*	1.9	2.6	0.2	9.3
		*Cyanobiaceae*	4.7	11.9	0.4	42.5
		*SAR116 clade*	0.26	0.52	0.02	1.9
		*Cryomorphaceae*	0.85	2.2	0.08	7.7

RA = Relative abundance.

**Table 3:** Core microbiomes by genotype disease susceptibility categories in *A. cervicornis*. Percentages were generated by aggregating ASVs at the family level. ASVs that were not classified to the family level are listed to the lowest taxonomic classification.

Species	Susceptibility	Taxon	%Mean RA	%SD RA	%Min RA	%Max RA
*Acropora cervicornis*	Low (N = 18; C14, C24, C30)	*Midichloriaceae*	93.2	21.8	5.9	99.8
		*P3OB-42*	4.3	17.0	0.01	72.1
		*Endozoicomonadaceae*	1.3	1.7	0.001	5.2
		*Helicobacteraceae*	1.0	3.6	0.008	15.3
		*Cyanobiaceae*	0.3	0.7	0.005	3.00
	Mid (N = 28; C18, C20, C28, C29)	*Midichloriaceae*	88.0	27.7	6.3	99.9
		*P3OB-42*	10.0	27.3	0.008	91.5
		*Endozoicomonadaceae*	1.9	2.7	0.02	0.2
		Proteobacteria	0.04	0.04	0.009	0.2
	High (N = 14; C17, C21, C22)	*Midichloriaceae*	95.5	4.9	82.8	99.3
		*Endozoicomonadaceae*	2.8	4.4	0.05	15.4
		*P3OB-42*	1.2	1.9	0.01	6.8
		*Helicobacteraceae*	0.3	0.4	0.06	1.4
		*Spirochaetaceae*	0.08	0.03	0.05	0.1
		*Cyanobiaceae*	0.1	0.09	0.02	0.3
		Proteobacteria	0.04	0.01	0.004	0.06

RA = Relative abundance, C# = *Acropora cervicornis* genotype designation.

## Discussion

In this study, we evaluated the microbiomes of nursery-reared Caribbean *Acropora* corals that were tested for disease susceptibility^21^. In the grafting assays, inoculated *Acropora* were visually identified as diseased because definitive disease diagnostics do not exist for coral. Thus, the inoculated corals may have been presented with distinct pathogens. With the use of 16S rRNA gene high-throughput sequencing we showed that: (1) across all *A. palmata*, alpha-diversity was significantly higher compared to all *A. cervicornis*, (2) across all samples of *A. cervicornis* and *A. palmata*, microbial communities were distinct between the two species, (3) both coral species showed different dominant bacteria independent of treatment (4) grafting assays, resulted in a unique microbiome pattern in each coral species, (5) both coral species may have been infected by *Sphingomonadaceae*, and (6) in both coral species a core bacterium may have a role in disease susceptibility.

The microbiomes of healthy *A. cervicornis* and *A. palmata* were recently shown to harbor distinct microbial communities^38^ and our results further support this study (Fig. 1). Relative to other corals species, Caribbean acroporids have more similar microbial communities^38,39^, which is interesting since each *Acropora* spp. in our study harbored predominantly distinct microbes (Fig. 1D). Across *A. cervicornis* genotypes assessed, the bacterial family with the highest relative abundances was *Midichloriaceae* from the order Rickettsiales (Fig. 1D). This finding confirms past studies that describe high abundances of this family in apparently healthy *A. cervicornis* colonies^7,40,41^. The relationship between *Midichloriaceae* and *A. cervicornis* may be complicated because Rickettsiales has also been linked to disease^40,42,43^ and may take nutrients from *A. cervicornis*^44^. In a study of *A. cervicornis* in Panama, the authors did not find *Midichloriaceae* at high abundances but instead found *Endozoicomonadaceae* and *Campylobacteraceae* as the dominant bacterial taxa^39^. These differences in dominant bacteria in *A. cervicornis* may be due to different methods used, environmental factors, or differences between genotypes. Future baseline microbiome studies on multiple *A. cervicornis* genotypes across regions could help decipher these inconsistencies in the literature.

In *A. palmata*, we identified the families *Spirochaetaceae* and *Endozoicomonadaceae* as the dominant bacteria (Fig. 1D). To our knowledge, *Spirochaetaceae* has not been reported in high abundance in *A. palmata*^39,45^. These inconsistencies across studies, again, may be due to differences in methods, region, or differences between genotype microbiome composition. Our results show that *A. palmata* genotypes can have disparate dominant bacterial taxa; for example, genotype “P7”, had a higher relative abundance of an uncultured bacterium and *Endozoicomonadaceae* and had significantly lower abundances of *Spirochaetaceae* compared to the other five genotypes (Supplemental Figs. 3B and 1D). These differences in microbial communities may affect the way specific genotypes battle disease exposure.

In both *Acropora spp*., exposure to diseased ramets shifted the microbiome, but the pattern was unique for each coral species (Fig. 2). In *A. palmata*, inoculation resulted in shifts in the microbiome from a distinct healthy cluster to a distinct diseased cluster (Fig. 2C). This shift resulted in a similar dispersion of beta-diversity across healthy and diseased samples (Fig. 2D). However, *A. cervicornis* corals showed a different pattern to inoculations that aligns with the Anna Karenina Principle–in which the beta-diversity of diseased samples does not form a distinct cluster but rather undergoes a stochastic change^46^. In *A. cervicornis* the microbial community showed this stochastic change in a “halo^46^” pattern around the cluster of healthy samples (Fig. 2A). This resulted in control corals with a lower microbiome dispersion and beta-diversity, and inoculated corals with higher microbiome dispersion and beta-diversity (Fig. 2B) B). The distinct microbiome patterns to disease exposure of each coral species may be due to underlying mechanisms utilized for pathogen control. For instance, genotypes from *A. cervicornis* showed lower risks of developing tissue loss than did *A. palmata* genotypes^21^, which may be partially attributed to their microbial community patterns.

The etiological agent(s) transmitted to test ramets is (are) unknown, but based on histological analysis it was presumed to have caused rapid tissue loss (RTL) in some of the coral ramets^21^. We found four ASVs from the families *Vibrionaceae, Sphingomonadaceae, Rhodobacteraceae*, and *Cryomorphaceae* that were significantly associated with inoculation outcomes (Fig. 3). A single ASV, designated *Sphingobium yanoikuyae* (family *Sphingomonadacea*), was significantly detected in both species of disease-exposed samples. *S. yanoikuyae* was found in 67% of diseased samples, 54% of visually unaffected samples (corals that were inoculated with a disease but showed no sign of tissue loss) and one control sample (Fig. 3C). Our results suggest that corals from the disease-challenge assays may have generally been inoculated by the same pathogen. However, because we did not find *S. yanoikuyae* in 100% of diseased samples, it may be possible that not all corals were inoculated with the same pathogen(s), and this may explain some of the variations in disease susceptibility, especially within genotype^21^. It is also possible that these corals were inoculated by the same pathogen, but we were unable to identify the disease agent(s) with our methods. For instance, the pathogen(s) may have been viral, eukaryotic, or fungal, but we only examined bacteria and archaea signatures. In addition, coral ramets were sampled at the base, which resulted in samples with varying distances from the disease margin. Sampling the lesion margin directly may have resulted in increased detection of *S. yanoikuyae* and/or other microbial members related to the disease.

If the disease-causing agent was bacterial and infected all samples, we would expect the pathogen(s) to be present in all of the diseased samples. The core microbiome analysis of diseased samples did not result in any taxa that were found in >99% of diseased samples, and that were nearly absent or at low relative abundances in control samples. We did identify an ASV from the family *Cyanobiaceae* as part of the disease-core microbiome of both species (Table 2), but this ASV was also found in the majority of control samples at similar relative abundances to diseased samples. Thus, we believe that if the disease agent was bacterial then the inoculants were unlikely to have harbored the same pathogens. In contrast, *S. yanoikuyae* was only found in one control sample at a relative abundance below the 25th percentile of inoculated samples (Fig. 3A,B). The genus *Sphingobium* has been associated with heat-stressed corals^47^, white plague in *Diploria strigosa*^48^, and unusual lesions in *Porites astreoides*^49^, but it has not been reported as a putative coral pathogen. The presence of *S. yanoikuyae* in both coral species across 67% of diseased corals and its presence in only one control coral suggests that this bacterium may be a putative coral pathogen. The relatively low abundances of *S. yanoikuyae* implies that coral pathogens may not always be the most abundant members in a diseased host’s microbial community. This observation has been noted in other studies and is highlighted by the keystone-pathogen hypothesis^50^. However, higher relative abundances may have been reported if samples were taken near the lesion.

The three other significant taxa (*Vibrionaceae, Rhodobacteraceae*, and *Cryomorphaceae)* that we identified at relatively high abundances in inoculated corals have been linked to coral diseases^42,51^. A similar study found that *A. cervicornis* inoculated with WBD homogenates also resulted in significant shifts in *Vibrionaceae, Rhodobacteraceae*, and *Cryomorphaceae*. The researchers concluded that *Rhodobacteraceae* and *Cryomorphaceae* were likely opportunistic and *Vibrionaceae* were unlikely primary pathogens^42^. In our results, the *Cryomorphaceae* ASV was found in 100% of diseased and visually unaffected *A. palmata* samples (Fig. 3C and Table 2) but was not present in *A. cervicornis*. Given that all inoculant ramets were from the species *A. cervicornis* it seems unlikely that the pathogen would not be found in any diseased *A. cervicornis*. Since *Vibrionaceae, Rhodobacteraceae*, and *Cryomorphaceae* did not significantly change in both species across the treatment, we also conclude that they are unlikely primary pathogens and are instead opportunist or secondary pathogens.

Samples that were exposed to disease but remained visually unaffected imply that these corals were resilient to the inoculant. Visually unaffected samples exposed to disease had similar bacterial signatures to those corals with tissue loss (Fig. 3); although phenotypically they appeared healthy. It is possible that these corals might have developed signs of disease if the experiment was conducted during a longer time-frame (i.e., >7 d). In spite of this, the ability for these corals to appear visually unaffected in contrast to corals that developed tissue loss suggests that they may have an underlying mechanism of resistance. A similar conclusion was reached in a WBD challenge experiment^42^.

Patterns of resilience were evident based on genotype^21^, but also independent of genotype. For example, although all *A. palmata* genotypes were categorized as relatively highly susceptible to disease^21^, there were still *A. palmata* ramets that were inoculated with disease but remained visually unaffected. We characterized the core microbiome to identify possible vital microbes^37^ in samples that remained visually unaffected and that may have unique microbial signatures independent of genotype. The core microbiome of visually unaffected *A. cervicornis* samples did not have any unique ASVs compared to control and diseased samples (Table 2). However, the high relative abundance of Myxococcales (14.2%, family *P3OB-42;* Table 2) in visually unaffected samples could mean they have some importance in combating disease. The core microbiome of visually unaffected *A. palmata* had 4 unique ASVs compared to control and diseased samples (from the families *Vibrionaceae, Phycisphaeraceae*, and *P3OB-42* and a Proteobacterium). From these taxa, Proteobacterium and *P3OB-42* had the highest relative abundances in visually unaffected samples (Table 2). The ASV from the family *Vibrionaceae* was different from the *Vibrio* from the ANCOM results (Fig. 3). *Vibrio* spp., are common marine pathogens, symbionts, commensals, and opportunists^52^, and were identified as part of the core microbiome of the surface mucus of apparently healthy *Acropora granulosa* corals^53^. Hence, the presence of a *Vibrio* spp. as a core microbial member in visually unaffected corals highlights the difficulties in distinguishing whether these are beneficial or pathogenic microbes. Future investigations into the role of *Vibrio* as a core microbial member will help us to understand its complicated function in coral health.

In the 2017 grafting experiment^21^ there were no *A. palmata* genotypes categorized as disease resistant, with all tested genotypes showing 70–90% risk of transmission. This observation is further supported by our results that did not find a significant interaction between treatment-genotype or outcome-genotype. Contrarily, in 2017, three *A. cervicornis* genotypes (C17, C21, and C22) were categorized as susceptible (or highly susceptible; >89% Risk of transmission) to disease^21^. Our results, also found that there was a significant interaction between outcome and genotype in *A. cervicornis*. Thus, there may be a genotype-specific microbiome response against disease. In the core microbiome analysis of relatively high-, mid-, and low disease-susceptible corals, the ASVs were shared throughout these categories (Table 3). However, we did find *P3OB-42* at relatively higher abundances in low (4.3%) and mid (10.0%) samples compared to highly (1.6%) susceptible corals (Table 3).

The presence of the order Myxococcales (*P3OB-42*) in the *Acropora* core microbiome of corals categorized as visually unaffected and disease-resistant genotypes is compelling. These bacteria are predators that have been associated with other coral core microbial predatory bacteria^54^ and they have also been shown to have co-evolved with corals^55^. The role of the order Myxococcales has not been investigated thoroughly in corals but based on our results it may be an interesting order to explore for its role (if any) in pathogen control.

## Conclusion

In this study, we aimed to identify putative pathogens and patterns associated with disease susceptibility from a disease-challenge experiment. We identified potential putative and opportunistic and/or secondary pathogens of *A. cervicornis* and *A. palmata*. We concluded that the bacterial families *Vibrionaceae, Rhodobacteraceae*, and *Cryomorphaceae* were unlikely primary pathogens. Although, we hypothesize that these are not primary pathogens, this does not negate their importance in coral disease. The constant presence of these families in coral disease studies make these potential targets for disease mitigation and merit further research. We also conclude that the family *Sphingomonadaceae* may be a putative pathogen since this bacterium were present in 67% of diseased corals. We also identified a core microbiome ASV at relatively high abundances, from the order Myxococcale*s*, that was associated with visually unaffected corals and genotypes with relatively low/mid disease-susceptibility. Further investigations, such as culturing and testing if direct inoculation with Myxococcales reduces disease development or progression of pathogens in corals can help elucidate if this bacterium plays a role in disease resistance.

## Supplementary information

Revision1

## References

[1] Lirman D, Schopmeyer S (2016). Ecological solutions to reef degradation: optimizing coral reef restoration in the Caribbean and Western Atlantic. PeerJ.

[2] Schopmeyer SA (2012). *In Situ* Coral Nurseries Serve as Genetic Repositories for Coral Reef Restoration after an Extreme Cold-Water Event. Restoration Ecology.

[3] Rinkevich B (1995). Restoration Strategies for Coral Reefs Damaged by Recreational Activities: The Use of Sexual and Asexual Recruits. Restoration Ecology.

[4] Lirman D (2010). Propagation of the threatened staghorn coral Acropora cervicornis: methods to minimize the impacts of fragment collection and maximize production. Coral Reefs.

[5] Lirman D (2014). Growth Dynamics of the Threatened Caribbean Staghorn Coral *Acropora cervicornis*: Influence of Host Genotype, Symbiont Identity, Colony Size, and Environmental Setting. PLoS ONE.

[6] Ladd M, Shantz A, Bartels E, Burkepile D (2017). Thermal stress reveals a genotype-specific tradeoff between growth and tissue loss in restored Acropora cervicornis. Marine Ecology Progress Series.

[7] Miller MW, Lohr KE, Cameron CM, Williams DE, Peters EC (2014). Disease dynamics and potential mitigation among restored and wild staghorn coral, *Acropora cervicornis*. PeerJ.

[8] Porter JW, Meier OW (1992). Quantification of Loss and Change in Floridian Reef Coral Populations. American Zoologist.

[9] Precht W, Bruckner A, Aronson R, Bruckner R (2002). Endangered acroporid corals of the Caribbean. Coral Reefs.

[10] Aronson, R. & Precht, W. *Aronson RB, Precht WF. White-band disease and the changing face of Caribbean coral reefs. Hydrobiologia 460: 25–38*. vol. 460 (2001).

[11] Hoegh-Guldberg O (2007). Coral Reefs Under Rapid Climate Change and Ocean Acidification. Science.

[12] Harvell CD (2002). Climate Warming and Disease Risks for Terrestrial and Marine Biota. Science.

[13] Vollmer SV, Kline DI (2008). Natural Disease Resistance in Threatened Staghorn Corals. PLoS ONE.

[14] Muller, E. M., Bartels, E. & Baums, I. B. Bleaching causes loss of disease resistance within the threatened coral species *Acropora cervicornis*. *eLife* **7** (2018). 10.7554/eLife.35066 PMC6133546 30203745

[15] Wright, R. M. *et al*. Intraspecific differences in molecular stress responses and coral pathobiome contribute to mortality under bacterial challenge in *Acropora millepora*. *Scientific Reports* **7** (2017). 10.1038/s41598-017-02685-1 PMC5454005 28572677

[16] Peixoto, R. S. *et al*. Beneficial Microorganisms for Corals (BMC): Proposed Mechanisms for Coral Health and Resilience. *Frontiers in Microbiology* **8** (2017). 10.3389/fmicb.2017.00341 PMC5339234 28326066

[17] Bourne DG, Morrow KM, Webster NS (2016). Insights into the Coral Microbiome: Underpinning the Health and Resilience of Reef Ecosystems. Annual Review of Microbiology.

[18] Fragoso ados Santos, H. *et al*. Impact of oil spills on coral reefs can be reduced by bioremediation using probiotic microbiota. *Scientific Reports* **5** (2016). 10.1038/srep18268 PMC4677405 26658023

[19] Welsh RM (2017). Alien vs. predator: bacterial challenge alters coral microbiomes unless controlled by *Halobacteriovorax* predators. PeerJ.

[20] Rosado PM (2019). Marine probiotics: increasing coral resistance to bleaching through microbiome manipulation. The ISME Journal.

[21] Miller MW (2019). Genotypic variation in disease susceptibility among cultured stocks of elkhorn and staghorn corals. PeerJ.

[22] Bythell John, Pantos Olga, Richardson Laurie (2004). White Plague, White Band, and Other “White” Diseases. Coral Health and Disease.

[23] Sweet MJ, Croquer A, Bythell JC (2014). Experimental antibiotic treatment identifies potential pathogens of white band disease in the endangered Caribbean coral *Acropora cervicornis*. Proceedings of the Royal Society B: Biological Sciences.

[24] Miller, M. W. & Williams, D. E. A standard field protocol for testing relative disease resistance in *Acropora palmata* and *Acropora cervicornis*., 10.7287/peerj.preprints.2668v1.

[25] Apprill A, McNally S, Parsons R, Weber L (2015). Minor revision to V4 region SSU rRNA 806R gene primer greatly increases detection of SAR11 bacterioplankton. Aquatic Microbial Ecology.

[26] Gilbert, J. A., Jansson, J. K. & Knight, R. The Earth Microbiome project: successes and aspirations. *BMC Biology* **12** (2014). 10.1186/s12915-014-0069-1 PMC4141107 25184604

[27] Bolyen, E. *et al*. QIIME 2: Reproducible, interactive, scalable, and extensible microbiome data science., 10.7287/peerj.preprints.27295v2. 10.1038/s41587-019-0209-9 PMC7015180 31341288

[28] Martin M (2011). Cutadapt removes adapter sequences from high-throughput sequencing reads. EMBnet. journal.

[29] Callahan BJ (2016). DADA2: High-resolution sample inference from Illumina amplicon data. Nature Methods.

[30] Altschul SF, Gish W, Miller W, Myers EW, Lipman DJ (1990). Basic local alignment search tool. Journal of Molecular Biology.

[31] McMurdie PJ, Holmes S (2013). phyloseq: An R Package for Reproducible Interactive Analysis and Graphics of Microbiome Census Data. PLoS ONE.

[32] Leo, L. *et al*. *Tools for microbiome analysis in R*. *Microbiome package version*. (2017).

[33] Dixon P (2003). VEGAN, a package of R functions for community ecology. Journal of Vegetation Science.

[34] Clarke KR (1993). Non-parametric multivariate analyses of changes in community structure. Austral Ecology.

[35] Mandal, S. *et al*. Analysis of composition of microbiomes: a novel method for studying microbial composition. *Microbial Ecology in Health & Disease* **26** (2015). 10.3402/mehd.v26.27663 PMC4450248 26028277

[36] Turnbaugh PJ, Gordon JI (2009). The core gut microbiome, energy balance and obesity: The core gut microbiome, energy balance and obesity. The Journal of Physiology.

[37] Hernandez-Agreda A, Gates RD, Ainsworth TD (2017). Defining the Core Microbiome in Corals’ Microbial Soup. Trends in Microbiology.

[38] Dunphy, C. M., Gouhier, T. C., Chu, N. D. & Vollmer, S. V. Structure and stability of the coral microbiome in space and time. *Scientific Reports* **9** (2019). 10.1038/s41598-019-43268-6 PMC6494856 31043671

[39] Chu ND, Vollmer SV (2016). Caribbean corals house shared and host-specific microbial symbionts over time and space: Specificity and overlap in coral microbiomes. Environmental Microbiology Reports.

[40] Casas V (2004). Widespread association of a Rickettsiales-like bacterium with reef-building corals. Environmental Microbiology.

[41] Godoy-Vitorino F, Ruiz-Diaz CP, Rivera-Seda A, Ramírez-Lugo JS, Toledo-Hernández C (2017). The microbial biosphere of the coral *Acropora cervicornis* in Northeastern Puerto Rico. PeerJ.

[42] Gignoux-Wolfsohn, S. A., Aronson, F. M. & Vollmer, S. V. Complex interactions between potentially pathogenic, opportunistic, and resident bacteria emerge during infection on a reef-building coral. *FEMS Microbiology Ecology* **93** (2017). 10.1093/femsec/fix080 28637338

[43] Shaver EC (2017). Effects of predation and nutrient enrichment on the success and microbiome of a foundational coral. Ecology.

[44] Klinges JG (2019). Phylogenetic, genomic, and biogeographic characterization of a novel and ubiquitous marine invertebrate-associated Rickettsiales parasite, Candidatus Aquarickettsia rohweri, gen. nov., sp. nov. The ISME Journal.

[45] Beltrán Y (2016). Microbial composition of biofilms associated with lithifying rubble of *Acropora palmata* branches. FEMS Microbiology Ecology.

[46] Zaneveld JR, McMinds R, Vega Thurber R (2017). Stress and stability: applying the Anna Karenina principle to animal microbiomes. *Nature*. Microbiology.

[47] Littman RA, Bourne DG, Willis BL (2010). Responses of coral-associated bacterial communities to heat stress differ with *Symbiodinium* type on the same coral host. Molecular Ecology.

[48] Cárdenas A, Rodriguez-R LM, Pizarro V, Cadavid LF, Arévalo-Ferro C (2012). Shifts in bacterial communities of two caribbean reef-building coral species affected by white plague disease. The ISME Journal.

[49] Meyer JL, Paul VJ, Teplitski M (2014). Community Shifts in the Surface Microbiomes of the Coral Porites astreoides with Unusual Lesions. PLoS ONE.

[50] Hajishengallis G, Darveau RP, Curtis MA (2012). The keystone-pathogen hypothesis. Nature Reviews Microbiology.

[51] Gignoux-Wolfsohn SA, Vollmer SV (2015). Identification of Candidate Coral Pathogens on White Band Disease-Infected Staghorn Coral. PLOS ONE.

[52] Thompson FL, Iida T, Swings J (2004). Biodiversity of Vibrios. Microbiology and Molecular Biology Reviews.

[53] D Ainsworth T (2015). The coral core microbiome identifies rare bacterial taxa as ubiquitous endosymbionts. The ISME Journal.

[54] Welsh RM (2016). Bacterial predation in a marine host-associated microbiome. The ISME Journal.

[55] Pollock, F. J. *et al*. Coral-associated bacteria demonstrate phylosymbiosis and cophylogeny. *Nature Communications* **9** (2018). 10.1038/s41467-018-07275-x PMC6250698 30467310

