# Supplementary material for: Microbiome differences in disease-resistant vs. susceptible Acropora corals subjected to disease challenge assays
Source: Sci Rep. 2019 Dec 4;9:18279. doi: 10.1038/s41598-019-54855-y (PMC6892807; doi:10.1038/s41598-019-54855-y)
Supplement: Supplementary file 1 — Revision1 [file 41598_2019_54855_MOESM1_ESM.pdf]

Title: Microbiome differences in disease-resistant vs. susceptible *Acropora* corals subjected to disease challenge assays

Authors: Stephanie M. Rosales [1,2]*, Margaret W. Miller[3], Dana Williams[4], Nikki Traylor-Knowles[5], Ben Young[5], Xaymara M. Serrano[1,2]

Affiliations:

1. Atlantic Oceanographic and Meteorological Laboratory, National Oceanographic and Atmospheric Administration, Miami, Florida, USA
2. Cooperative Institute for Marine and Atmospheric Studies, University of Miami, Miami, Florida, USA
3. SECORE International, Miami FL 33145
4. Southeast Fisheries Science Center, NOAA-National Marine Fisheries Service, Miami, FL, USA
5. University of Miami, Rosenstiel School of Marine and Atmospheric Sciences

* Correspondence Author email: Stephanie.Rosales@noaa.gov

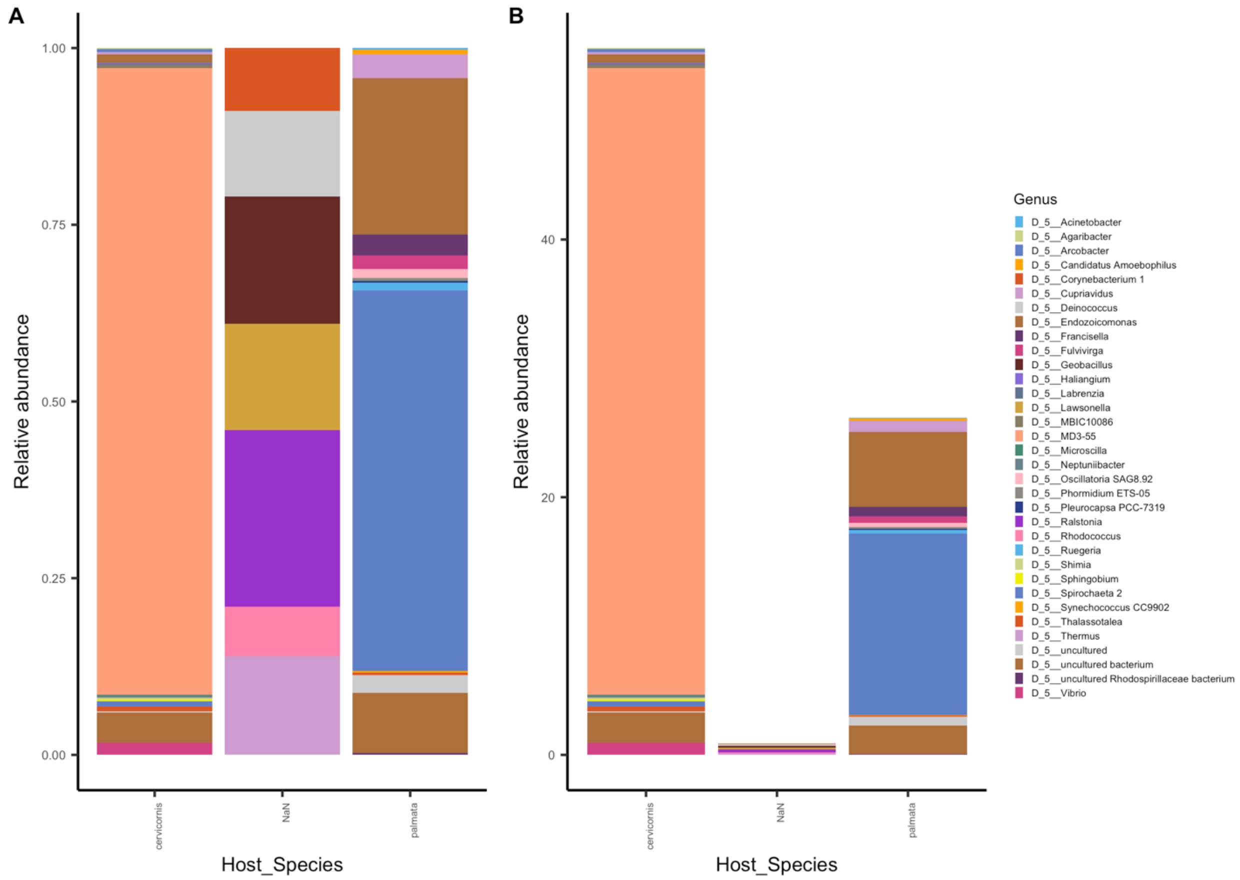

Supplemental Fig. 1. Negative PCR control shows distinct microbial members from coral samples and at low relative abundances. The percent relative abundances of the most abundant microbial genera (> 0.05%, not rarefied) of each host species and negative control (NaN). Each stacked color bar represents a different genus in (A) cumulative proportions and (B) average relative abundance.

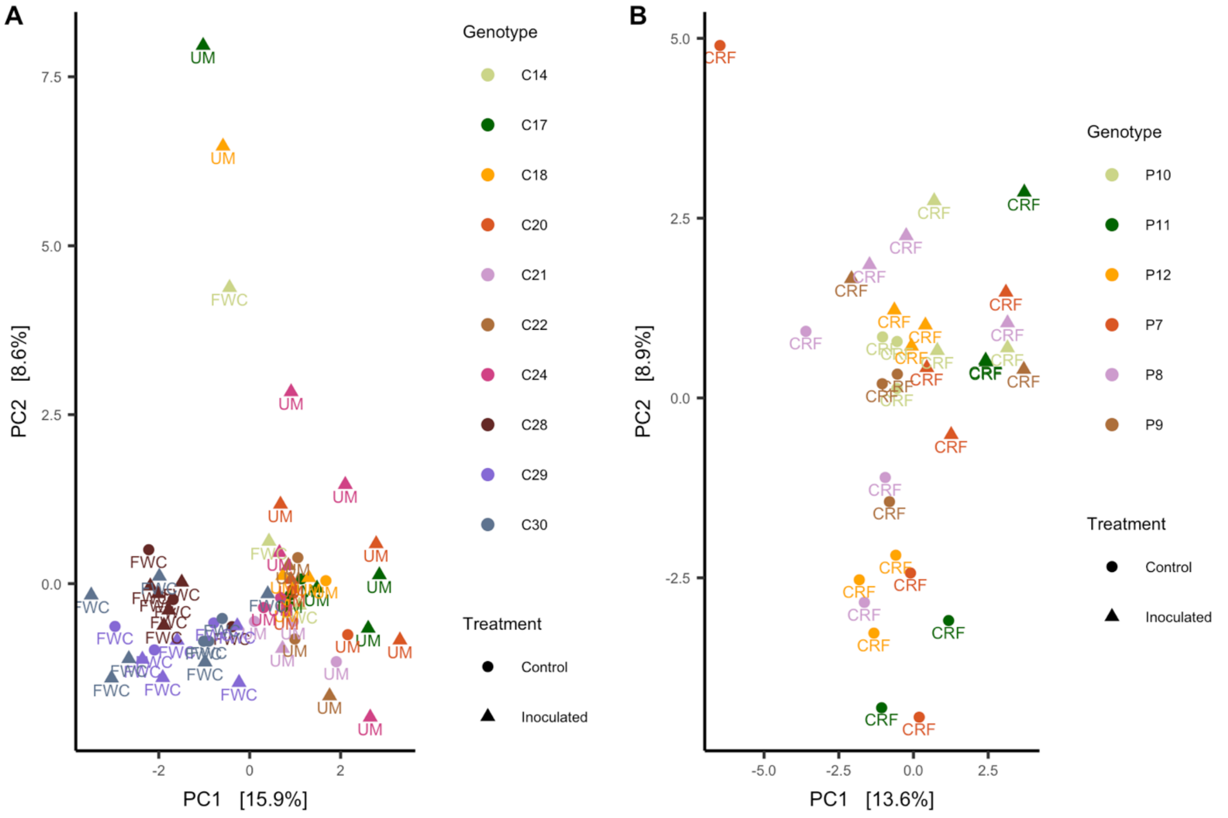

Supplemental Fig. 2. Microbial beta-diversity of coral *A. cervicornis* and *A. palmata* from disease-challenge experiments. Principal component analysis (PCA) with a Euclidean distance of (A) *A. cervicornis* and (B) *A. palmata* colored by genotype, shaped by treatment, and labeled by nursery where samples were reared.

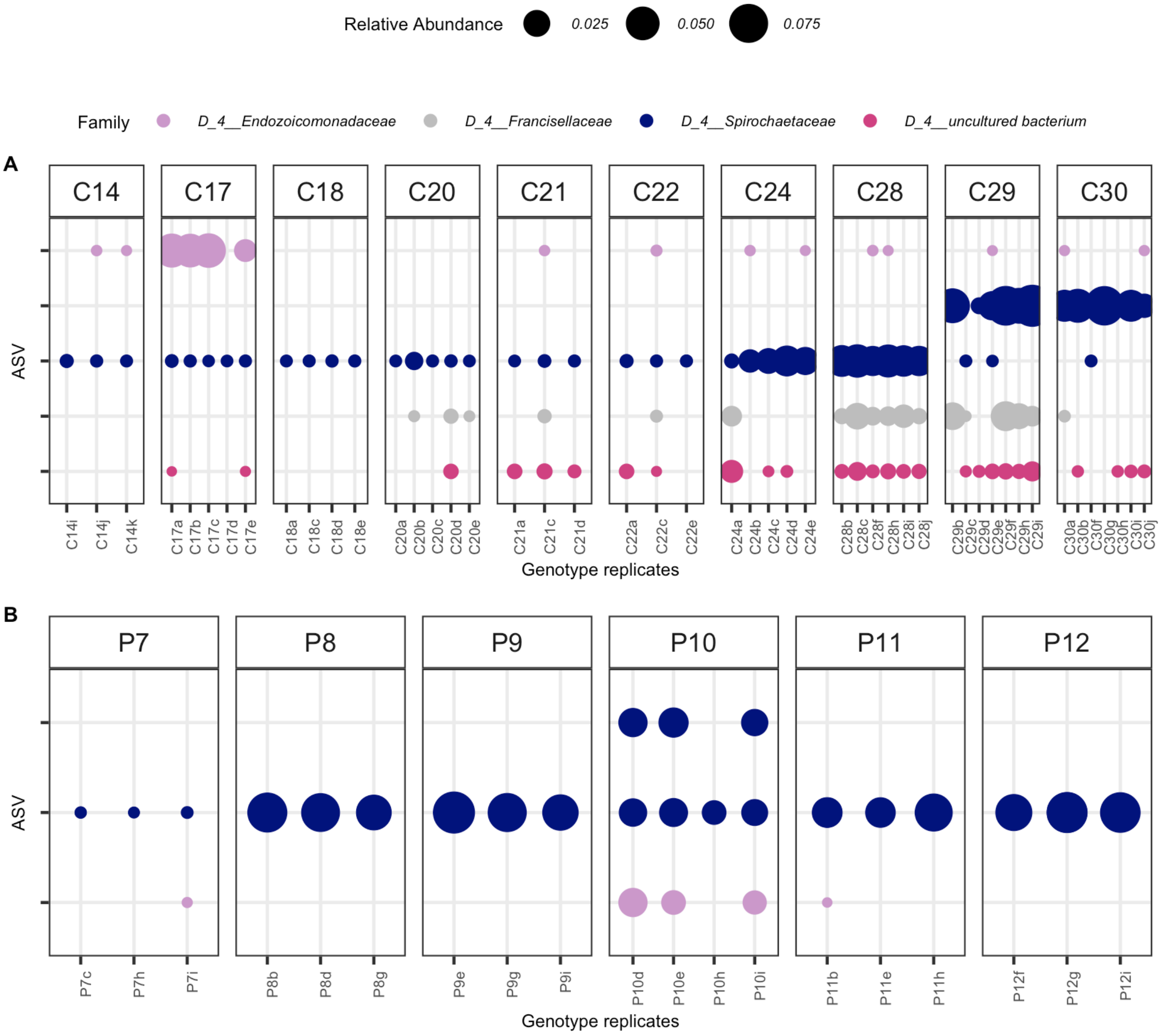

Supplemental Fig. 3. Differential abundance analysis across genotypes. (A) ASVs that were significantly differentially abundant across the different genotypes in (A) *A. cervicornis* and (B) *A. palmata.* Dots are colored by bacteria family and size is based on relative abundance of each ASV in a sample. Only samples which had the significant differential abundant ASVs are shown in each plot.
